# Physicochemical Properties and Biological Activities of Silver Carp Scale Peptide and Its Nanofiltration Fractions

**DOI:** 10.3389/fnut.2021.812443

**Published:** 2022-01-04

**Authors:** Xiao-yan Zu, Ya-jing Zhao, Shi-ming Fu, Tao Liao, Hai-lan Li, Guang-quan Xiong

**Affiliations:** ^1^Institute of Agricultural Products Processing and Nuclear Agricultural Technology, Hubei Academy of Agricultural Sciences, Wuhan, China; ^2^College of Petrochemical, Lanzhou University of Technology, Lanzhou, China

**Keywords:** silver carp scale peptide, physicochemical properties, free radical scavenging, tyrosinase inhibition, amino acid sequence

## Abstract

To explore the physicochemical properties and biological functions of silver carp scale peptide (SCSP), its molecular-weight fractions SCSP-I, II, and III obtained by nanofiltration were assessed for their solubility, emulsibility, free radical scavenging ability, effect on the proliferation of mouse B16 cells. The results showed that the solubility of each fraction of SCSP was higher than 90%, SCSP-II and III were higher than 95%. The antioxidant powers on ⦁OH, ${\text{O}}_{2}^{-}$⦁ and Fe^3+^ were ranked as SCSP-III > SCSP-II > SCSP-I > SCSP. All fractions of SCSP had no toxic or side effects in mouse B16 melanoma cells experiments *in vitro*. At a concentration of 0.01 mg/mL, the tyrosinase activity of B16 cells in the SCSP-II fraction was significantly lower than that of the α-arbutin (*P* < 0.05), at 65.37%. The molecular weight distribution of SCSP was 399–1404 Dalton and 13 peptide sequences were detected. Among them, SCSP-II contained many hydrophobic amino acids, and SCSP-III stood out for combining arginine with hydrophobic amino acids. This may be the reason why the low molecular-weight SCSPs show the strong antioxidant activity and strong tyrosinase inhibition. The work provides a data base for the development of SCSP and increases the possibility of its application.

## Introduction

Silver carp (*Hypophthalmichthys molitrix*) is widely distributed in lakes and rivers across Asia. A fast-growing fish, it has high feed efficiency and high nutritional value, so it is one of the four major farmed freshwater fishes in China. In 2020, the aquaculture production of silver carp reached 3.81 million tons, second only to grass carp (Ministry of Agriculture Fishery Administration., 2021). During the processing of silver carp, many by-products are produced, including fish heads, bones, skins, and internal organs. Their weight accounts for approximately 50–70% of the total body weight of the fish, of which fish scales account for approximately 5% (1). For a long time, fish scales have been considered of low value, so the rate of fish scale utilization and processing has been low, resulting in the serious waste of biological resources.

The high-quality collagen that is enriched in fish scales can undergo the disintegration of molecular chains *via* hydrolysis, forming peptide mixtures between proteins and amino acids (2). Fish scale peptide is characterized by high solubility, good thermal stability, and easy absorption (3). And it can have multiple biological functions such as binding to mineral ions (4), inhibiting aging and antioxidation (5). Addition, according to Ma's conclusion that short peptides containing 2–10 amino acids have greater antioxidant potential and biological activity than their native proteins or peptides (6). The composition and proportion of hydrophobic amino acids (e.g., Pro, Ala, Val, Phe, Leu, Ile) in the sequence are also related to the antioxidant activity of polypeptides (7), the presence of hydrophobic amino acid residues can enhance the antioxidant capability of peptides, and the higher their content, the stronger the antioxidant activity (8).

Excellent biocompatibility and low immunogenicity enable the fish scale peptides to be used in biomedical fields, such as wound healing and medical dressings. Due to its strong antioxidant, moisturizing, and whitening properties, fish scale peptide has been approved for uses in cosmetics, health foods, and other products (9, 10). At present, the overuse of popular skin-whitening agents on the market, such as arbutin and hydroquinone, can cause permanent white patches on the skin (11). Therefore, it is imperative to find a safe and effective natural inhibitor of melanin synthesis.

In this paper, the physicochemical properties of silver carp scale peptide (SCSP) and its three molecular-weight fractions were studied. The amino acid sequences and molecular weights of the corresponding peptides were identified by mass spectrometry. Free radical scavenging assays, *in vitro* cell experiments, and a tyrosinase inhibition assay were carried out to verify their antioxidant and whitening effects. This study is expected to provide technical support for the development of SCSP as a consumer product.

## Materials and Methods

### Materials

Silver carp scales were purchased from Liangzihu Aquatic Products Processing Co., Ltd. (Wuhan, China). Soybean oil (food grade) was from the COFCO Fulinmen Food Marketing Co., Ltd. (Wuhan, China). Alkaline protease (liquid, 2,400,000 U/mL) was from Genencor (China) Bioengineering Co., Ltd. 2, 2-Diphenyl-1-picrylhydrazyl (DPPH) was from Sigma Corporation (USA). B16-F1 mouse melanoma cells were from the China Center for Type Culture Collection (CCTCC, Hubei, Wuhan). 3-(4, 5-Dimethylthiazol-2-yl)-2, 5-diphenyltetrazolium bromide (MTT) was purchased from BioFroxx (Germany). Trypsin-EDTA (0.25%), fetal bovine serum, and penicillin-streptomycin were from GIBCO (New York, USA). Chromatographically pure acetonitrile was from Thermo Fisher Scientific Co., Ltd. (Shanghai, China). All other reagents used in the experiment were of analytically pure grade and were from Sinopharm Chemical Reagent Co., Ltd. (Shanghai, China).

### Technical Route

**Figure 1:**
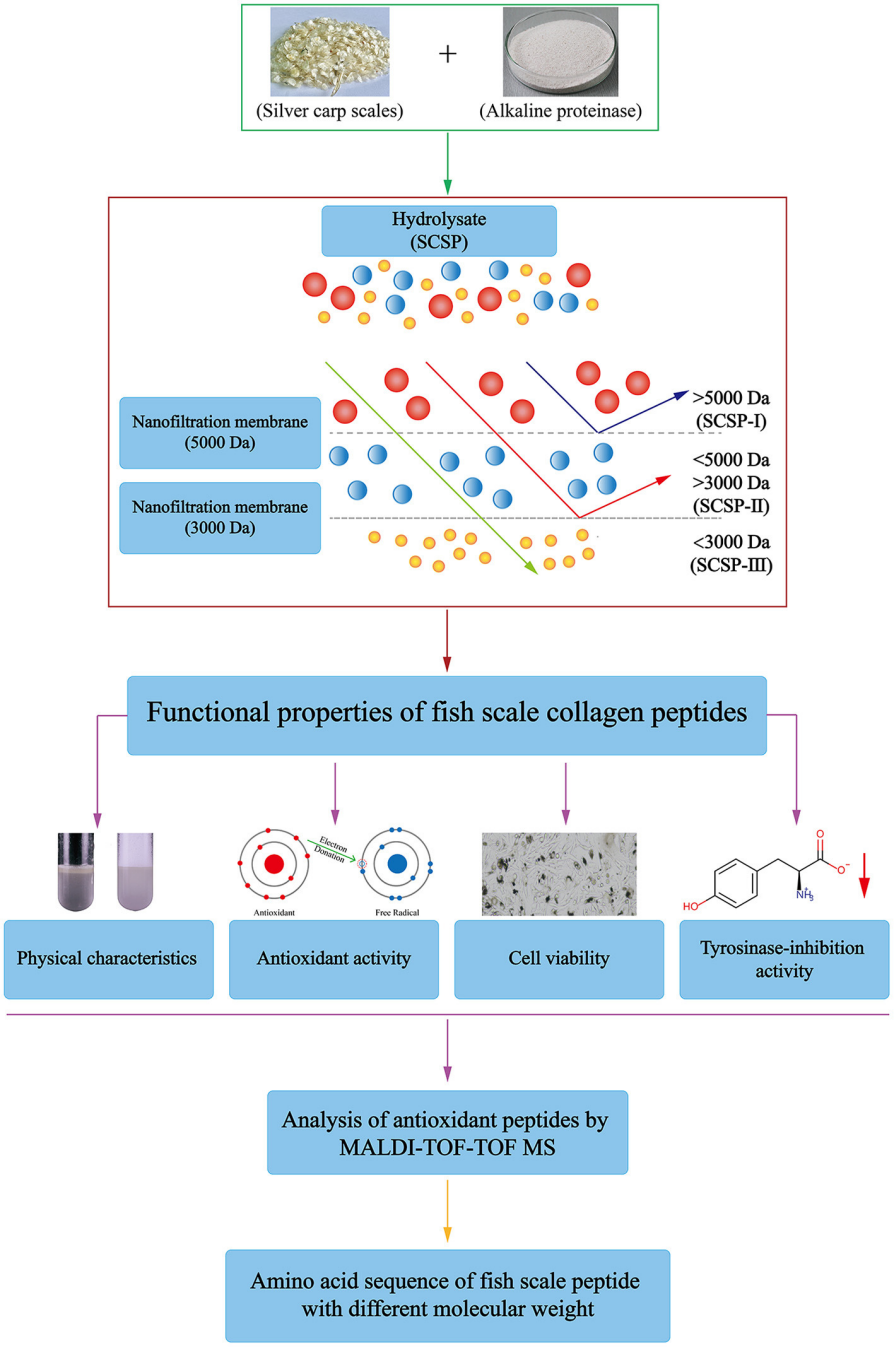
Technical roadmap for the preparation and analysis of SCSPs.

In this study, decalcified silver carp scales were hydrolyzed with alkaline protease to obtain an enzymatic hydrolysate. After removing the enzymes, the enzymatic hydrolysate was separated by nanofiltration to prepare different molecular-weight fractions of SCSP. The physicochemical properties and biological activities of SCSP and its three molecular-weight fractions were studied. The technical flow chart is shown in Figure 1.

### Preparation of SCSP and Its Nanofiltration Fractions

The decalcified silver carp scales were cleaned with deionized water to remove impurities and mixed with deionized water at a ratio of 1:50 (w/v), and 0.01 M sodium hydroxide (NaOH) was used to adjust the solution pH to 8.0. Then alkaline protease was added, and the mixture was mixed evenly and placed at 50°C to undergo enzymatic hydrolysis until all the solid scales had disappeared. The enzymatic hydrolysate was inactivated at 100°C for 10–15 min and then centrifuged at 5,000 rpm for 20 min to remove the impurities and enzymes to obtain the SCSP.

The SCSP was filtrated and separated with a nanofiltration membrane (S-UF3, Shanghai Langji membrane separation equipment Engineering Co., Ltd., China) with a molecular weight cut-off of 5,000 Dalton (Da), and the filtrate continued to be separated by a nanofiltration membrane with a molecular weight cut-off of 3,000 Da. The substances retained by the 5,000-Da nanofiltration membrane were the SCSP-I fraction, the substances retained by the 3000-Da nanofiltration membrane were the SCSP-II fraction, and the filtrate that passed through the 3,000-Da nanofiltration membrane was the SCSP-III fraction. The SCSP sample before nanofiltration and different molecular-weight fractions were freeze-dried for later use.

### Determination of Solubility

The method of Adler-Nissen et al. (12) was used as a reference for the determination of collagen peptide solubility, with slight modifications. In 50 mL of distilled water, 5 mg of the freeze-dried SCSP, SCSP-I, SCSP-II, and SCSP-III samples were dissolved, and the pH values were, respectively, adjusted to 3.0, 4.0, 5.0, 6.0, 7.0 8.0, 9.0, and 10.0 with 6.0 M HCl or 6.0 M NaOH solution, followed by centrifugation at 4,000 rpm for 20 min. In 1 mL of the supernatant, the biuret method was used to determine the polypeptide contents. The solubility of SCSP and its fractions was calculated according to Equation (1), and the result is expressed as the nitrogen solubility index (NSI):


(1) $$\begin{array}{l} \text{NSI (}\%\text{)}=\frac{\text{N1}}{\text{N2}}\times \text{100}\% \end{array}$$

where N_1_ is the polypeptide content in the supernatant, g/g; and N_2_ is the total polypeptide content in the sample, g/g.

### Determination of Emulsibility and Emulsifying Stability

Emulsibility was measured as described by Vioque et al. (13) with slight modifications. In 40 mL of distilled water, 2 g of SCSP, SCSP-I, SCSP-II, or SCSP-III freeze-dried powder was dissolved, and its pH was adjusted to 7.0, followed by adding 10 mL of soybean oil. The sample was homogenized in a high-speed homogenizer (FJ3000, Shanghai Specimen and Model Factory, China) for 2 min, the homogenized mixture was centrifuged at 1,000 rpm for 5 min, and the emulsifying activity index (EAI) was calculated according to Equation (2). After the measurement, the centrifuge tube was placed in a constant-temperature water bath (HH-6, Changzhou Zhiborui Instruments Manufacturing Co., Ltd., China) at 50°C for 30 min. After cooling to room temperature, the sample was centrifuged at 1,500 rpm for 10 min. According to Equation (3), the emulsifying stability index (ESI) was calculated.


(2) $$\begin{array}{l} \text{EAI (}\%\text{)}=\frac{\text{H1}}{\text{H}}\times \text{100}\% \end{array}$$

(3) $$\begin{array}{l} \text{ESI (}\%\text{)}=\frac{\text{H2}}{\text{H1}}\times \text{100}\% \end{array}$$

where H is the total height of the liquid in the centrifuge tube, cm; H_1_ is the height of the emulsion layer, cm; and H_2_ is the height of the emulsion layer after centrifugation, cm.

### Measurement of Antioxidant Activity

The freeze-dried powders of SCSP, SCSP-I, SCSP-II, and SCSP-III were added to deionized water to prepare sample solutions of 1, 2, 4, 6, 8, and 10 mg/mL for the measurement of antioxidant activity.

#### Measurement of DPPH⦁ Scavenging Activity

The method of Bougatef et al. (14) with slight modifications was used to measure DPPH⦁ scavenging activity. In a test tube, 2.5 mL of SCSP, SCSP-I, SCSP-II, or SCSP-III sample solution of different concentrations was added, followed by adding 2.5 mL of DPPH solution (0.1 mmol/L) prepared in absolute ethanol. The mixture was shaken for 10 s and then left to react in the dark at room temperature for 30 min. Next, the absorbance of the solution was measured at 517 nm with an ultraviolet-visible spectrophotometer (UH5300, Hitachi High-Technologies Corporation, Japan). The blank control had 2.5 mL of distilled water. The DPPH radical scavenging rate of the sample solution was calculated according to Equation (4):


(4) $$\begin{array}{l} \text{DPPH radical scavenging activity (}\%\text{)}=\frac{\text{A0}-\text{A}}{\text{A0}}\times \text{100}\% \end{array}$$

where A_0_ represents the absorbance of the DPPH**⦁** ethanol solution without sample and A represents the absorbance of the DPPH**⦁** ethanol solution in the measured sample.

#### Measurement of ⦁OH Scavenging Activity

The method of Fu et al. (15) was used to determine the capability of SCSP to scavenge **⦁**OH. In an Erlenmeyer flask, 1 mL of SCSP, SCSP-I, SCSP-II, or SCSP-III sample solution of different concentrations was added with a pipette, followed by adding 1 mL salicylic acid-ethanol solution (10 mmol/L), 1 mL FeSO_4_ solution (10 mmol/L), 2 mL H_2_O, and 5 mL H_2_O_2_ solution (10 mmol/L). The flask stood at 37°C for 15 min. The absorbance of the solution was measured at 510 nm with an ultraviolet-visible spectrophotometer. The hydroxyl radical scavenging rate of the sample solution was calculated according to Equation (5):


(5) $$\begin{array}{l} \text{OH radical scavenging activity (}\%\text{)}=\frac{\text{A0}-\text{A}}{\text{A0}}\times \text{100}\% \end{array}$$

where A_0_ represents the background absorbance and A represents the absorbance of the measured sample.

#### Measurement of Ferric-Reducing/Antioxidant Power

The method of Bougatef et al. (14) was used to measure the capability of the SCSP to reduce Fe^3+^. After 1 mL of SCSP, SCSP-I, SCSP-II, or SCSP-III sample solution of different concentrations was mixed evenly with 1 mL (1%) K_3_[Fe(CN)_6_] and 1 mL (0.2 mol/L) phosphate-buffered saline (PBS) buffer solution, the mixture was reacted in a constant-temperature water bath at 50°C for 20 min. After it had cooled to room temperature, 1 mL (10%) trichloroacetic acid solution was added to consume excess K_3_[Fe(CN)_6_], and the sample was centrifuged at 4,000 rpm for 10 min. To 2.5 mL of the supernatant, 0.75 mL (0.1%) FeCl_3_ solution and 2.5 mL of distilled water were added, the mixture was placed in a constant-temperature water bath at 50°C for 10 min until Prussian blue appears. Then the absorbance of the solution was measured at 700 nm.

#### Measurement of ${\text{O}}_{2}^{-}$ Radical Scavenging Activity

The method of Wang et al. (16) with slight modifications was used to determine the ${\text{O}}_{2}^{-}$⦁ scavenging activity. In an Erlenmeyer flask, 4.2 mL of different concentrations of SCSP, SCSP-I, SCSP-II, or SCSP-III sample solution was added by pipette, followed by adding 4.5 mL of Tris-HCl (0.1 mol/L, pH 8.2) buffer solution. The sample was mixed evenly and let stand in water bath at 25°C for 15 min, followed by quickly adding 0.3 mL (3 mmol/L) of pyrogallol (prepared with 10 mmol/L HCl). Then, the solution was mixed evenly. Distilled water instead of peptide solution served as the blank control. The absorbance was measured at 325 nm every 30 s for 4 min. The slope of absorbance over time was calculated. The ${\text{O}}_{2}^{-}$⦁ scavenging rate of the solution was calculated according to Equation (6):


(6) $$\begin{array}{l} \text{Superoxide anion radical scavenging activity (}\%\text{)}\\ \;=\frac{\text{k0}-\text{k}}{\text{k}}\times \text{100}\% \end{array}$$

where k_0_ represents the slope of the blank group and k represents the slope of the sample group.

### Cell Viability Determination by MTT Assay and Cell Morphology Observation

The method of Huang et al. was used to measure cell viability (17). After the B16 melanoma cells in the logarithmic growth phase were inoculated in 96-well plates at 1 × 10^4^ cells/well, they were cultured in an incubator (MCO-15AC, SANYO Electric Co., Ltd., Japan) with 5% CO_2_ at 37°C for 12 h, and then the medium was changed. Among them, 100 μL of sample (0.01, 0.1, 0.5, or 1.0 mg/mL) was added to the medium of the experimental group. The positive control group received an equal volume of α-arbutin at the same concentration, the blank group an equal volume of medium. After cultivation for 48 h, 20 μL of 0.5% MTT was added to each well, and the cultivation continued for 2 more h. The supernatant was collected, followed by adding 200 μL of dimethyl sulfoxide, and the mixture was shaken for 10 min. The absorbance at 490 nm was measured with a microplate reader (Fluoroskan, Thermo Fisher Scientific Co., Ltd., UK). The cell viability was calculated by Equation (7):


(7) $$\begin{array}{l} \text{Cell viability (}\%\text{)}=\frac{\text{A}}{\text{A0}}\times \text{100}\% \end{array}$$

where A is the measured absorbance of the positive control group or the experimental group and A_0_ is the absorbance of the blank group.

To observe the cell morphology, B16 melanoma cells in the logarithmic growth phase were cultured in a cell culture dish. After 12 h, the medium was changed, and 1.0 mg/mL α-arbutin, SCSP, SCSP-I, SCSP-II, or SCSP-III sample solution was added. The cultivation continued for 48 more h, and then an inverted microscope (IX7, Olympus Co., Ltd., Japan) was used to observe and photograph the morphology of each group of cells.

### Determination of Tyrosinase Activity

The method of Huang et al. (17) was used to determine the tyrosinase activity. The cell inoculation method, inoculation density, and sample loading method were the same as those of section 2.7. After the cells had been cultured for 48 h, the cells were disrupted with 90 μL of PBS (pH 6.8) buffer containing 1% Triton X-100, and the resulting sample was centrifuged at 1,000 rpm for 30 min in a centrifuge. Considering the toxicity of high concentration α-arbutin, low concentration SCSPs of 0.01 mg/ml were selected for tyrosinase activity inhibition test. To 90 μL of cell disruption solution, 10 μL of 1 mg/mL l-3, 4-dihydroxyphenylalanine (l-DOPA) was added, and the sample was cultured at 37°C for 90 min. The absorbance at 475 nm was measured with a microplate reader. The intracellular tyrosinase activity was calculated by Equation (8):


(8) $$\begin{array}{l} \text{Tyrosinase activity (}\%\text{)}=\frac{\text{A}}{\text{A0}}\times \text{100}\% \end{array}$$

where A is the measured absorbance of the positive control group or the experimental group and A_0_ is the absorbance of the blank group.

### Determination of Molecular Weight

Matrix-assisted laser desorption ionization time-of-flight mass spectrometry (MALDI-TOF-MS) (autoflexmaX, Bruker Co., Ltd., Germany) was used to analyze the molecular weight and molecular weight distribution range corresponding to each SCSP, SCSP-I, SCSP-II, or SCSP-III sample. The sample was mixed with 2,5-dihydroxybenzoic acid (20 mg/mL) in 50% acetonitrile/water (containing 0.1% trifluoroacetic acid) at a ratio of 1:1. After 1 μL of the sample mixture was placed in one well of a 384-well plate and naturally dried at room temperature, the sample plate was placed in the ion source for measurement. The final mass spectrum was obtained by accumulating 10 single-scan signals.

### Determination of Amino Acid Sequence

The amino acid sequence of the peptides was determined by ultraperformance liquid chromatography-tandem mass spectrometry (UPLC-MS/MS) (Agilent6545Q-TOF, Waldbronn, Germany). The sequencing conditions were as follows: the sample volume was 5 μL, mobile phase A was 0.1% formic acid-acetonitrile, and mobile phase B was 0.1% formic acid-water. The gradient elution program was: 1–30% A (0–45 min), 30–90% A (45–50 min), 90% A (50–52 min), 90–1% A (52–55 min), and 1% A (55–60 min); the flow rate was 0.2 mL/min. Mass spectrometry conditions: the electrospray ion source was set to electrospray ionization positive ion scanning mode, the nebulizer and drying gas were high-purity nitrogen, the drying temperature was 200°C, the drying gas flow rate was 6.0 L/min. And the scanning range was controlled with the MS program with a mass-to-charge ratio (m/z) of 50–3000 and the MS/MS program with m/z = 50–2000. The data were analyzed using the Proteome Discoverer 2.4 software from Thermo Fisher (Thermo Fisher Scientific Co., Ltd., UK), and collagen peptides were classified by searching against the UniProt protein database (www.UniProt.org).

### Data Analysis

IBM SPSS Statistics 20.0 (International Business Machines Corporation Co., Ltd., USA) was used for statistical analysis. Differences with *P* < 0.05 were considered statistically significant. Origin 9.1 software (Origin Lab Co., Ltd., USA) was used to draw graphs. All experiments were carried out in triplicate, and the data are expressed as mean ± standard deviation.

## Results

### Solubility, Emulsibility, and Emulsifying Stability of SCSPs

**Figure 2:**
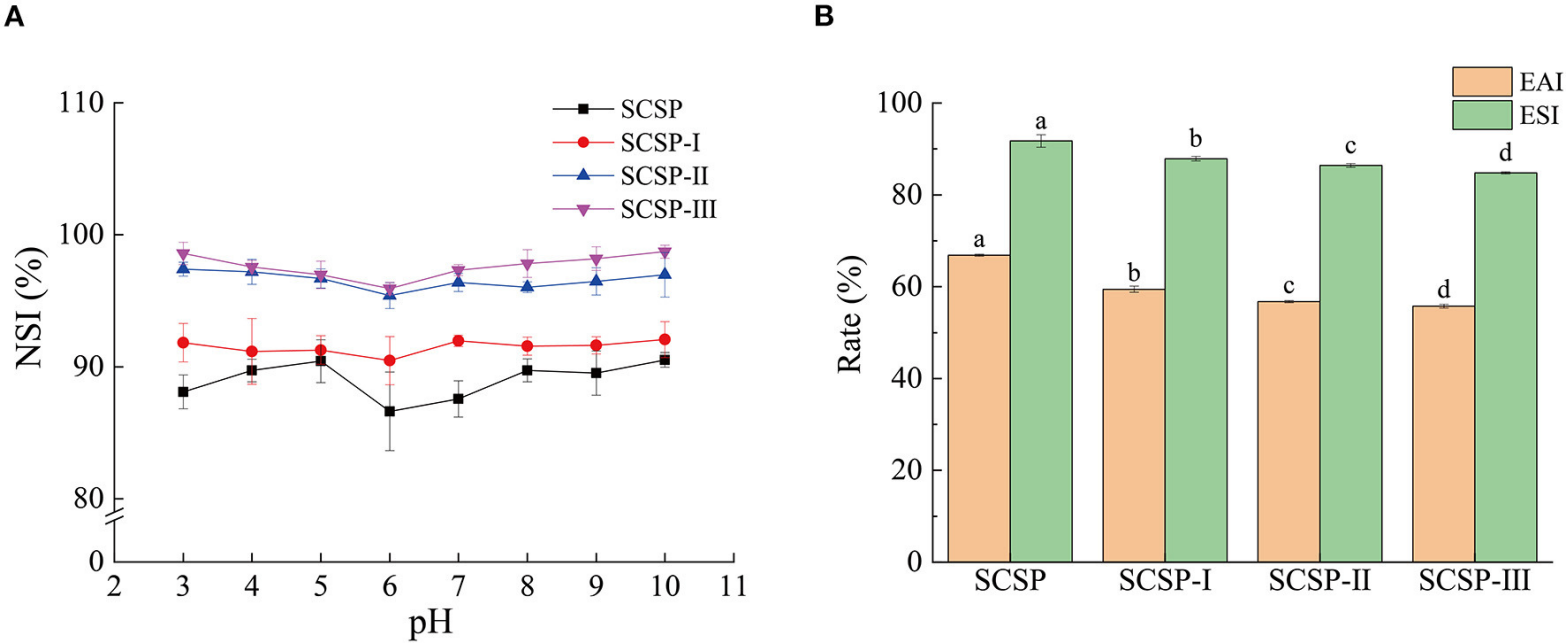
Solubility **(A)**, emulsibility and emulsifying stability **(B)** of SCSPs. The 0.1 mg/mL and 50 mg/mL SCSPs were used in the tests of solubility and emulsibility, respectively. Different lowercase letters denote significant differences between SCSP, SCSP-I, SCSP-II, and SCSP-III groups (*P* < 0.05). All experiments were repeated three times. Data are expressed as mean ± standard deviation.

As shown in Figure 2A, at different pH values, the solubilities of SCSP and its three fractions were ranked as SCSP-III > SCSP-II > SCSP-I > SCSP. The solubility of each fraction was higher than 90%, SCSP-III and SCSP-II having solubilities higher than 95%. In addition, under different pH values, each fraction showed a similar trend of changes in solubility. When the pH was <6, the solubility of each fraction decreased with increasing pH; when the pH was > 6, the solubility of each fraction slowly increased with increasing pH and eventually plateaued.

As shown in Figure 2B, the emulsibility and emulsifying stability significantly decreased with decreasing molecular weight (*P* < 0.05), emulsibility decreasing more significant than emulsifying stability. The emulsibility of SCSP was 66.89 ± 0.23%, and the emulsifying stability was as high as 91.74 ± 1.36% after the sample had stood for 30 min. The emulsibility and emulsifying stability of SCSP-III were only 55.77 ± 0.40% and 84.82 ± 0.23%, respectively.

### Antioxidant Effects of SCSPs

**Figure 3:**
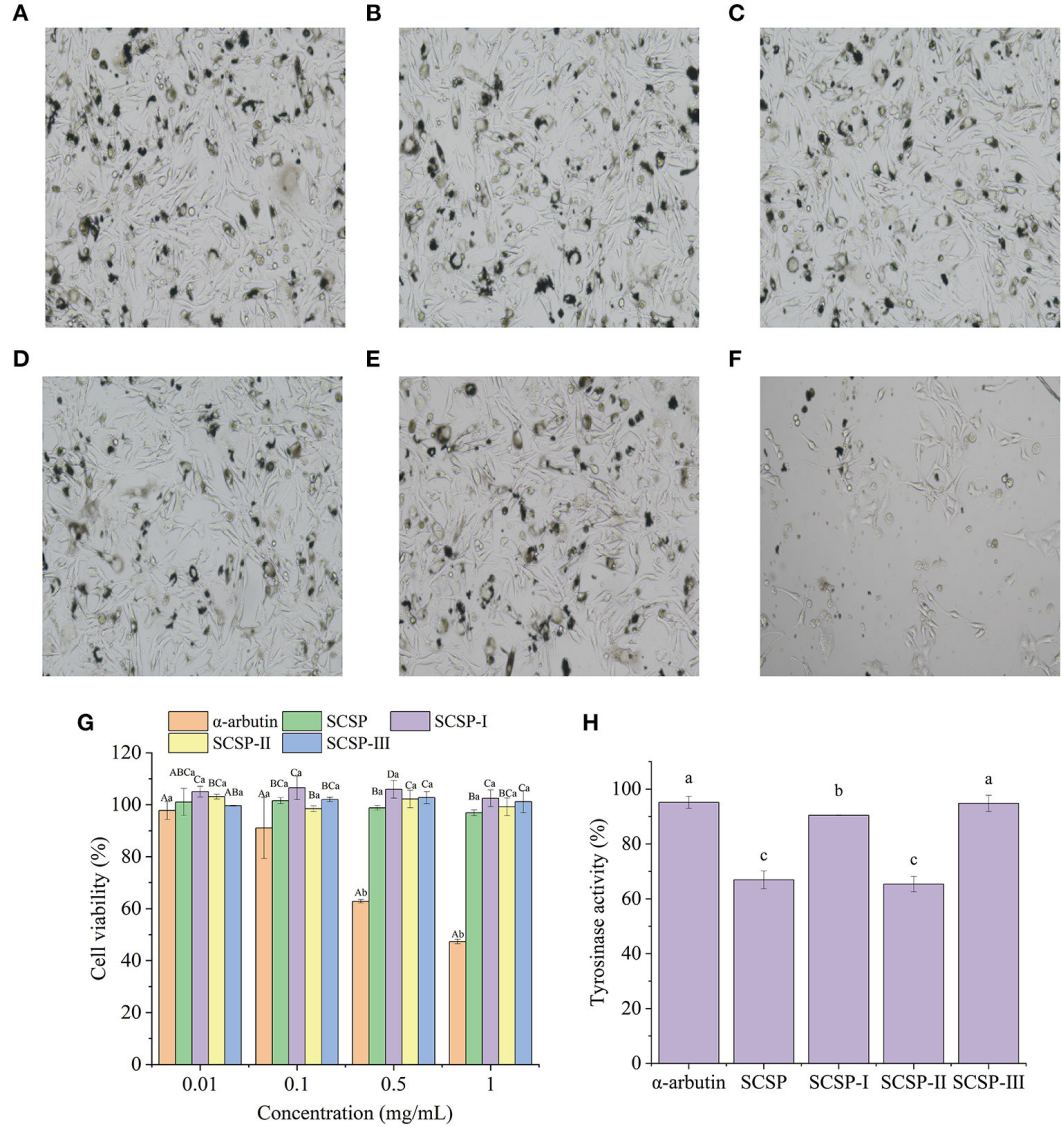
Morphology of mouse B16 melanoma cells in the normal state (blank control) **(A)**, and under the action of 1.0 mg/mL SCSP **(B)**, SCSP-I **(C)**, SCSP-II **(D)**, SCSP-III **(E)**, and α-arbutin **(F)**. The proliferative activity **(G)** of mouse B16 melanoma cells under the action of SCSPs at 0.01, 0.1, 0.5, 1.0 mg/mL, respectively. The cell tyrosinase activity **(H)** under the action of 0.01 mg/mL SCSPs. In **(G)**, different capital letters denote significant differences between the SCSP, SCSP-I, SCSP-II, and SCSP-III groups at the same concentration (*P* < 0.05), and different lowercase letters denote significant differences between the same fractions of different concentrations (*P* < 0.05). In **(H)**, different lowercase letters denote significant differences between groups (*P* < 0.05). All experiments were repeated three times. Data are expressed as mean ± standard deviation.

As shown in Figure 3A, in the range of 1–10 mg/mL, the DPPH⦁ scavenging rates of the SCSP and its three fractions all increased with increasing peptide concentration, and the increase in SCSP-I group was the most significant (*P* < 0.05). The ${\text{O}}_{2}^{-}$⦁ and ⦁OH scavenging rates and the ferric-reducing/antioxidant power of each fraction showed an upward trend with increasing peptide concentration (Figures 3B–D). At the same concentration, the fractions with lower molecular weights had higher antioxidant capability (Figures 3B–D).

### Effects of SCSPs on the Proliferation of Mouse B16melanoma Cells

**Figure 4:**
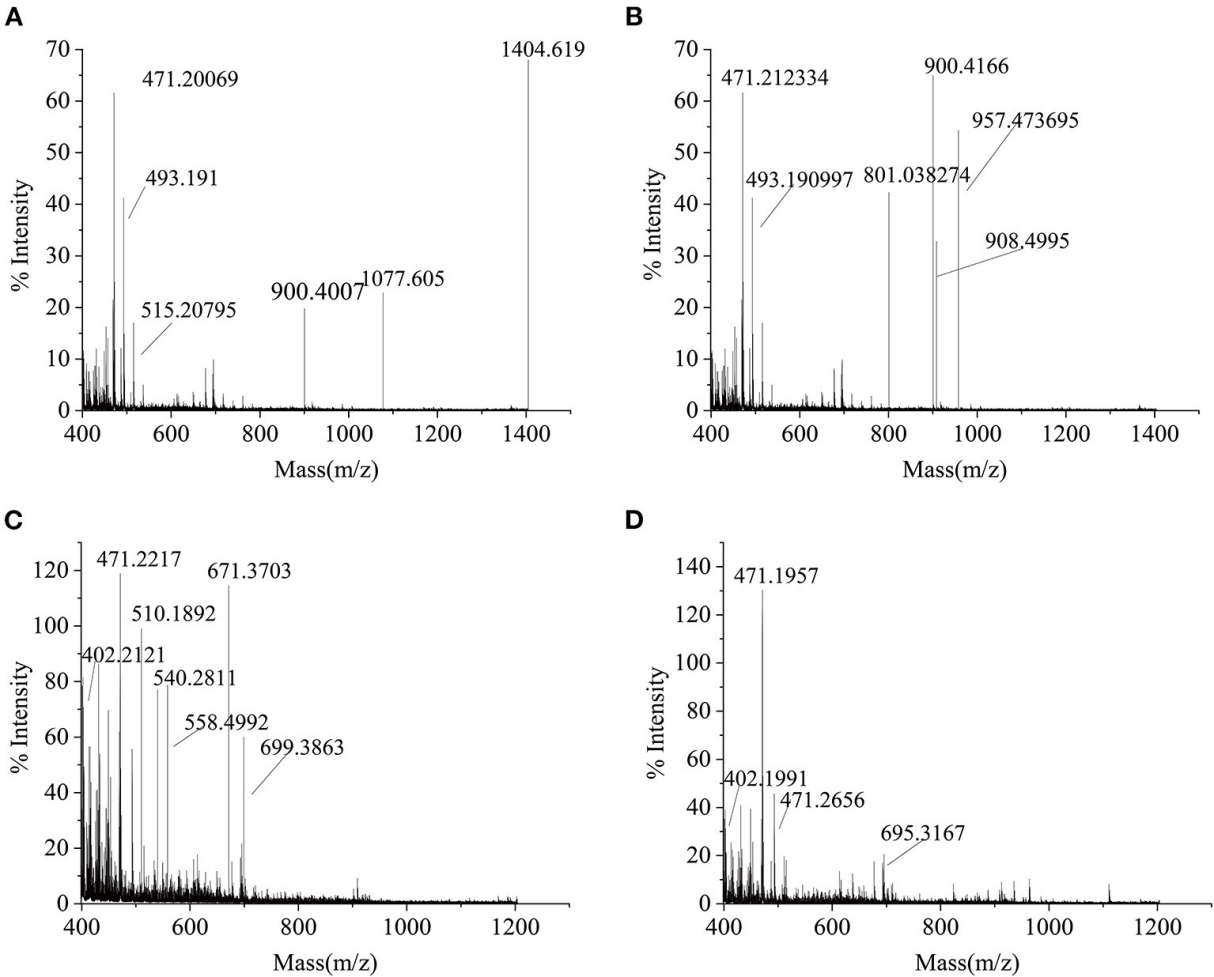
Antioxidant capability of SCSPs. **(A)** DPPH⦁ radical scavenging capability, **(B)** hydroxyl radical scavenging capability, **(C)** ferric-reducing/antioxidant power, **(D)** superoxide anion radical scavenging capability were measured under the action of SCSPs at 1, 2, 4, 6, 8, 10 mg/mL, respectively. Different capital letters denote significant differences between the SCSP, SCSP-I, SCSP-II, and SCSP-III groups at the same concentration (*P* < 0.05), and different lowercase letters denote significant differences between the same fractions with different concentrations (*P* < 0.05). All experiments were repeated three times. Data are expressed as mean ± standard deviation.

Figures 4A–F shows the morphology of mouse B16 melanoma cells under the action of 1.0 mg/mL sample. The cells in each SCSP group displayed uniform adhesion and were mostly spindle-shaped, with normal morphology, clear cell edges, and high cell density (Figures 4B–E). The SCSP groups were not significantly different from the blank control group (Figure 4A). In the α-arbutin group (Figure 4F), there were many apoptotic cells and no complete cell structure, the intercellular spaces became larger, dendritic structures disappeared, and there were fewer cells. The cell morphology results supported the results of the MTT cell viability assay. As shown in Figure 4G, except for α-arbutin, each sample had similar effects on the proliferation of mouse B16 melanoma cells as its concentration changed (*P* > 0.05). There was no significant difference in the proliferation of mouse B16 cells when the α-arbutin concentration was in the range of 0.01–0.1 mg/mL (*P* > 0.05). But when the α-arbutin concentration was higher than 0.1 mg/mL, it significantly inhibited B16 cell proliferation (*P* < 0.05), such that the cell survival rate was only 47.34 ± 0.88% in 1.0 mg/mL α-arbutin.

### Effects of SCSPs on the Tyrosinase Activity

Under the condition of 0.01 mg/mL, the effect of SCSPs on tyrosinase activity was studied with α-arbutin as positive control. As shown in Figure 4H, the inhibitory effect of each fraction on tyrosinase was higher than that of α-arbutin. Among them, the tyrosinase activity of the SCSP-II group was 65.37 ± 2.81%, which was significantly lower than that of the arbutin group (*P* < 0.05). Moreover, SCSP-II was seems to be safer to cells than α-arbutin. Tyrosinase is a key enzyme in the synthesis of melanin.

### Molecular Weight Ranges of SCSPs

**Figure 5:**
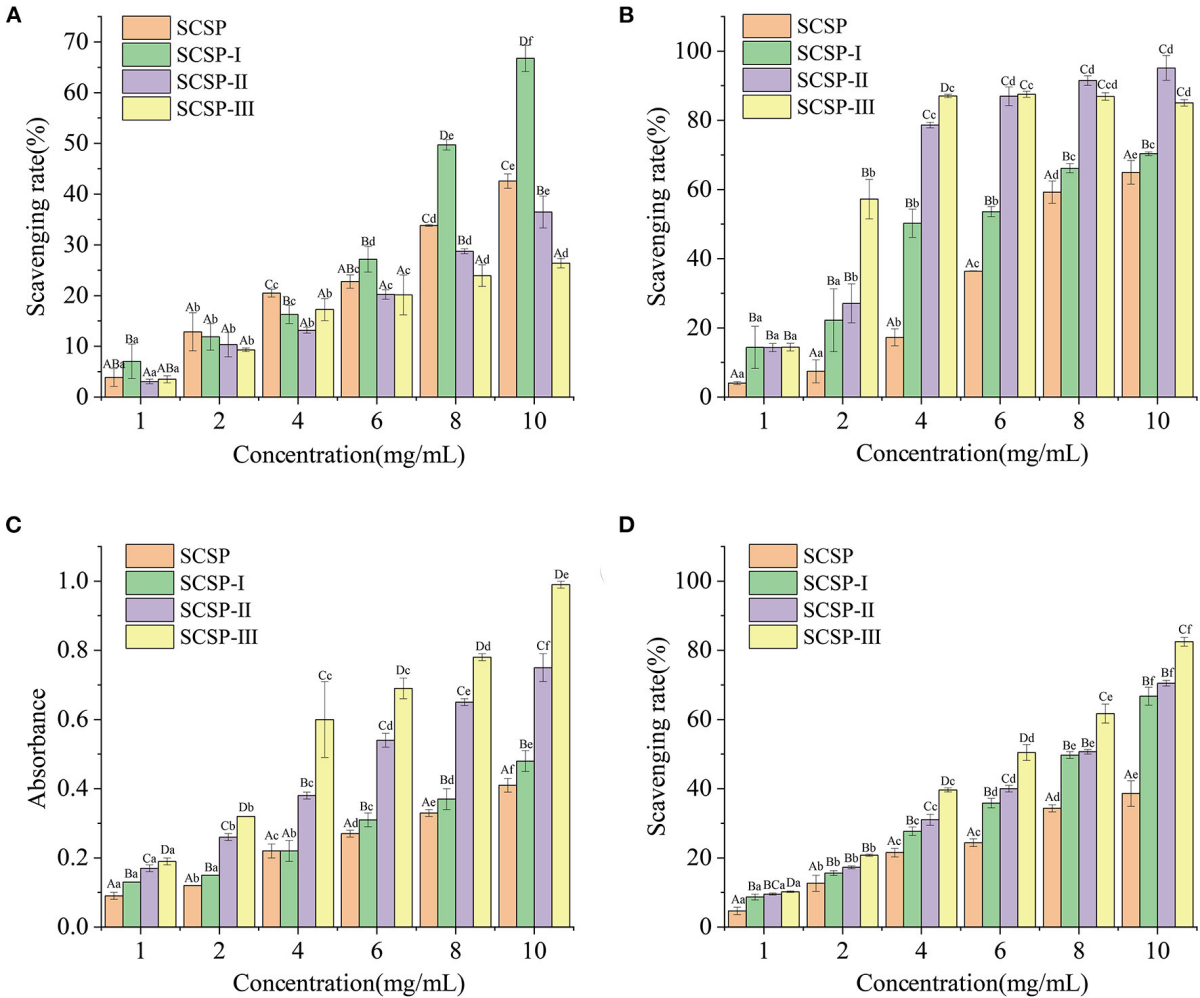
MALDI-TOF-MS spectral profiles of SCSPs. **(A)** SCSP, **(B)** SCSP-I, **(C)** SCSP-II, **(D)** SCSP-III.

As seen in Figure 5A, the molecular weight distribution of peptides in the SCSP ranged from 399 to 1404 Da, of which m/z 471.20 and 1404.62 were relatively abundant. Figure 5B shows that the molecular weight of SCSP-I ranged from 399 to 957 Da, of which m/z 471.21, and 900.42 were relatively abundant. Figure 5C shows that the molecular weight of SCSP-II ranged from 399 to 699 Da, of which m/z 471.22 and 671.37 were relatively abundant. Figure 5D shows that the molecular weight of SCSP-III ranged from 399 to 695 Da, in which m/z 471.20 were relatively abundant. The above results indicate that silver carp scales underwent complete enzymolysis under the action of alkaline protease.

### Amino Acid Sequence of SCSPs

As shown in Table 1, a total of 13 peptides were identified in SCSP. The peptides contained many Gly, Ala, and Pro, and Gly accounted for approximately 33.30% of the total sequence. These types of amino acids are essential amino acids for the synthesis of collagen (18). According to the molecular weight ranges of the peptides in Table 1, Figure 5, the obtained peptides were classified into the fractions SCSP-I, SCSP-II, and SCSP-III. The content of hydrophobic amino acids in the peptides GPAGPA, GPSGPA, and GIAGPA was generally higher than those of other peptides. Correspondingly, the antioxidant activity of SCSP-III was also higher than those of other SCSPs.

**Table 1:** The peptide sequence and molecular weight of SCSPs.

**No**.	**Sequence**	**Theo. MH+ [Da]**	**NF component**	**Confidence**	**XCor**
a	GARGDKGETGEA	1,147	SCSP-I	High	3.30
b	SGPAGPRGPAGSSGPA	1,322	SCSP-I	High	3.24
c	GMRGPRGA	801	SCSP-I	High	2.53
d	GERGEQGPA	900	SCSP-I	High	1.90
e	AGDPGPV	612	SCSP-II	High	1.86
f	VAGGIL	529	SCSP-II	High	1.62
g	RGDVGPA	671	SCSP-II	High	1.60
h	GRVGPA	556	SCSP-II	High	1.59
i	GAQGPIGA	670	SCSP-II	High	1.50
j	GNPGPA	512	SCSP-II	High	1.23
k	GPAGPA	469	SCSP-III	High	2.15
l	GPSGPA	485	SCSP-III	High	1.73
m	GIAGPA	485	SCSP-III	High	1.57

*The amino acids represented by capital letters in the table are as follows: A-Ala, G-Gly, R-Arg, D-Asp, K-Lys, E-Glu, S-Ser, M- Met, P-Pro, Q-Gln, V-Val, and I-Ile*.

## Discussion

### Physical Properties of SCSPs

According to the test results, the smaller the molecular weight of the peptide is, the higher the solubility is. The balance of hydrophilic forces and hydrophobic forces is a key factor affecting the solubility of peptides. Peptides with smaller molecular weights are expected to obtain more polar residues -COOH, -NH2, -OH, etc. (19). These hydrophilic groups can form hydrogen bonds with H2O and increase the force between peptide molecules and solvent molecules, thereby increasing the solubility of the peptide. With the increase of pH value, the solubility of the same SCSP first decreases and then increases and there seems to be a turning point at pH 6 (Figure 2A). This is because the solubility of the peptide also depends on the net charge of the molecule. The peptide solution near the isoelectric point is neutral, and the interaction between the solute and the solvent is stronger than the electrostatic effect, leading to aggregation and precipitation between the peptide solute molecules and a reduction of the solubility (20). When the pH is away from the isoelectric point, under the combined action of intermolecular force and electrostatic force, the peptide solute can be effectively dispersed in the solvent system, and the solubility increases. In the experiment, the emulsibility and emulsifying stability decreased with decreasing molecular weight (Figure 2B). This may be because the low intermolecular cross-linking of low-molecular-weight peptides is not enough to form a stable dispersion system, resulting in the decreases in emulsibility and emulsifying stability (21).

### Antioxidant Effects of SCSPs

On the case of DPPH results, there seems to be little correlations between DPPH clearance and peptide molecular weight, because that small-molecule peptides are mostly more polar and hydrophilic, making them less reactive with lipophilic DPPH⦁ free radicals (22). This difference may also have been related to the highest histidine mass fraction (0.14%) in SCSP-I. Because histidine is an important active site of antioxidant peptides, it can act as a proton donor to combine with DPPH⦁, thus improving the efficiency of SCSP-I at free radical scavenging (23). Figures 3B–D shows that the antioxidant powers were ranked as SCSP-III > SCSP-II > SCSP-I > SCSP, which was different from the ordering of DPPH⦁ scavenging rates. This may be because the polar molecules of low-molecular-weight peptides can provide many electrons, which can convert free radicals into stable products and terminate free-radical chain reactions (23, 24). Therefore, small-molecule peptides may exhibit better antioxidant properties in this respect.

### Biological Activities of SCSPs

An increase in melanin can lead to hyperpigmentation disorders, forming dark spots, spots, and freckles (25). Therefore, tyrosinase inhibitors, as an effective component to promote skin whitening, have received extensive attention in the field of cosmetics (26). Choi et al. have shown that low concentrations of fish scale peptide can be absorbed by cells and can effectively inhibit the tyrosinase activity in melanoma cells (27), which is supported by the results of this work. The α-arbutin produces toxic and side effects at high concentrations, blocking cell activity and causing cell apoptosis (28). In contrast, the fractions of SCSP were highly safe to mouse B16 cells even at high concentrations.

### Relations Between SCSP Biological Activity and Amino Acid Sequence

After enzymolysis, the resulting products were mostly small-molecule substances, and the signal intensity of small-molecule peptides after nanofiltration was significantly higher than that before nanofiltration, in line with the results of Cinq-Mars et al. (29). The primary structure and molecular weight of a peptide directly determines the strength of its antioxidant activity (21, 30).

In the work, the sequences of the SCSP-II and SCSP-III fractions were both shorter than 10 amino acids (Table 1), and compared with SCSP-I, the two had stronger antioxidant capability. These results are consistent with Ma's conclusion that short peptides have greater antioxidant activities (6). The content of hydrophobic amino acids in SCSP-III was higher than that of other SCSPs. This may be because hydrophobic amino acids act as hydrogen donors to interact with other amino acids to enhance the hydrophobic properties of the polypeptides (31), or act as hydrogen donors to react with metal ions and free radicals to reduce the oxidation rate (32), thereby enhancing their antioxidant capability.

The Gly, Ala, Ser, and Leu present in the fish scale peptides after nanofiltration can also enhance the peptides' tyrosinase inhibitory activity, thereby inhibiting the production of melanin and giving them a strong whitening effect (33). In addition, the combination of Arg or Phe residues and hydrophobic aliphatic residues (such as Val, Ala or Leu) can directly interact with tyrosinase to inhibit the formation of quinines (34). In RGDVGPA and GRVGPA of SCSP-II, Arg is combined with hydrophobic amino acids, which may be why SCSP-II showed strong tyrosinase inhibition (Figure 4H).

## Conclusions

The solubility of each component of SCSP was higher than 90%, and the smaller the molecular weight, the higher the solubility. In terms of antioxidation, generally, the lower the molecular weight of the peptide, the stronger the antioxidative activity. Among them, SCSP-III had a good scavenging effect on ⦁OH and ${\text{O}}_{2}^{-}$⦁ and had a strong ferric reducing/antioxidant power. Cell experiments *in vitro* showed that at a concentration of 0.01 mg/mL, SCSP-II had the strong inhibitory effect on tyrosinase, which was significantly better than that of α-arbutin. This peptide fraction was safe, exhibiting no toxic or side effects. The molecular weight distribution of SCSP was 399.83 ~ 1404.62 Da by MALDI-TOF-MS, and 13 peptide sequences were detected by UPLC-MS/MS. The peptides GPAGPA, GPSGPA, and GIAGPA, with relatively high contents of hydrophobic amino acids, may underlie the strong antioxidant activity of SCSP-III. The combination of Arg and hydrophobic amino acids in RGDVGPA and GRVGPA may be the reason why the SCSP-II component strongly inhibits tyrosinase. The above results indicate that short peptides SCSP-II and SCSP-III have good biological activity and can replace α-arbutin for melanin inhibition and oxidation inhibition. At present, although the SCSP shows the possibility of replacing α-arbutin as a whitening agent, but the product forms and sensory qualities (such as color, taste and smell) need to be further studied before application.

## Data Availability Statement

The original contributions presented in the study are included in the article/supplementary material, further inquiries can be directed to the corresponding authors.

## Author Contributions

XYZ: writing—original draft, formal analysis, methodology, investigation, and funding acquisition. YJZ: data curation, visualization, and writing—review and editing. SMF: visualization and writing—review and editing. TL: investigation and writing—review and editing. HLL: conceptualization, writing—review and editing, project administration, and funding acquisition. GQX: supervision, visualization, and writing—review and editing. All authors contributed to the article and approved the submitted version.

## Funding

This work was financed by the National Key Research and Development Program of China (2019YFD0902000).

## Conflict of Interest

The authors declare that the research was conducted in the absence of any commercial or financial relationships that could be construed as a potential conflict of interest.

## Publisher's Note

All claims expressed in this article are solely those of the authors and do not necessarily represent those of their affiliated organizations, or those of the publisher, the editors and the reviewers. Any product that may be evaluated in this article, or claim that may be made by its manufacturer, is not guaranteed or endorsed by the publisher.
